# CORUM in 2024: protein complexes as drug targets

**DOI:** 10.1093/nar/gkae1033

**Published:** 2024-11-11

**Authors:** Ralph Steinkamp, George Tsitsiridis, Barbara Brauner, Corinna Montrone, Gisela Fobo, Goar Frishman, Sorin Avram, Tudor I Oprea, Andreas Ruepp

**Affiliations:** Institute of Experimental Genetics, Helmholtz Zentrum München, German Research Center for Environmental Health (GmbH), Ingolstädter Landstr. 1, Neuherberg D-85764, Germany; Institute of Experimental Genetics, Helmholtz Zentrum München, German Research Center for Environmental Health (GmbH), Ingolstädter Landstr. 1, Neuherberg D-85764, Germany; Institute of Experimental Genetics, Helmholtz Zentrum München, German Research Center for Environmental Health (GmbH), Ingolstädter Landstr. 1, Neuherberg D-85764, Germany; Institute of Experimental Genetics, Helmholtz Zentrum München, German Research Center for Environmental Health (GmbH), Ingolstädter Landstr. 1, Neuherberg D-85764, Germany; Institute of Experimental Genetics, Helmholtz Zentrum München, German Research Center for Environmental Health (GmbH), Ingolstädter Landstr. 1, Neuherberg D-85764, Germany; Institute of Experimental Genetics, Helmholtz Zentrum München, German Research Center for Environmental Health (GmbH), Ingolstädter Landstr. 1, Neuherberg D-85764, Germany; Department of Computational Chemistry, “Coriolan Dragulescu” Institute of Chemistry, 24 Mihai Viteazu Blvd, Timisoara, Timis 300223, Romania; Expert Systems Inc., 12730 High Bluff Drive, San Diego, CA 92130, USA; Institute of Experimental Genetics, Helmholtz Zentrum München, German Research Center for Environmental Health (GmbH), Ingolstädter Landstr. 1, Neuherberg D-85764, Germany

## Abstract

CORUM (https://mips.helmholtz-muenchen.de/corum/) is a public database that offers comprehensive information about mammalian protein complexes, including their subunits, functions and associations with human diseases. The newly released CORUM 5.0, encompassing 7193 protein complexes, is the largest dataset of manually curated mammalian protein complexes publicly available. This update represents the most significant upgrade to the database in >15 years. At present, the molecular processes in cells that are influenced by drugs are only incompletely understood. In this latest release, we have begun systematically investigating the impact of drugs on protein complexes. Our studies are based on a dataset from DrugCentral comprising 725 protein drug targets with approved drugs and known mechanisms of action. To date, we have identified 1975 instances from the literature where a drug affects the formation and/or function of a protein complex. Numerous examples highlight the crucial role of understanding drug–protein complex relationships in drug efficacy. The expanded dataset and the inclusion of drug effects on protein complexes are expected to significantly enhance the utility and application potential of CORUM 5.0 in fields such as network medicine and pharmacological research.

## Graphical Abstract

**Graphical Abstract:**
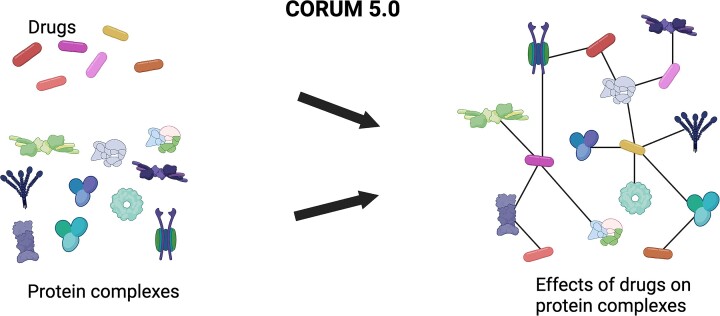

## Introduction

Major cellular processes such as the cell cycle, protein folding and ion transport depend on the activity of protein complexes. Therefore, a comprehensive understanding of the composition of complexes is essential for understanding cellular mechanisms. The CORUM database offers the largest publicly available collection of manually curated mammalian protein complexes, derived from experimental results in the literature. In recent years, the CORUM dataset has been widely used in numerous analyses as a reference for benchmarking computational models and high-throughput experimental data (1). Since its initial launch, the four CORUM publications have been cited >1900 times, according to Google Scholar (1–4).

Protein complexes are involved in a wide range of cellular processes and accordingly, dysfunction of complexes plays a key role in many human disorders. For example, Fanconi anaemia, a heterogeneous syndrome, involves seven genes that form a protein complex essential for DNA repair (5). As early as 2004, protein complexes were recognized as critical components in network medicine (6). Lage *et al.* conducted a large-scale analysis of the human phenome–interactome network, identifying protein complexes involved in genetic disorders (7). They scored candidate proteins based on the involvement of their direct network neighbours in similar diseases, suggesting potential protein complexes related to specific disorders. Vanunu *et al.* (8) developed a global network-based method for prioritizing disease genes and inferring protein complex associations. Using affinity purification–mass spectrometry methodology, Huttlin *et al.* (9) analysed protein communities and complexes, associating them with disease annotations. The importance of protein complexes and protein–protein interactions has also led to the development of small-molecule drugs designed to disrupt specific protein interactions. Notable examples include the KEAP1–NRF2 and VEGFA–NRP1 complexes (10).

A critical clinical issue is the heterogeneity of treatment effects, where the same treatment yields different results in different patients (11). Accordingly, a central goal of personalized medicine is to support more individualized clinical decision-making. Considering the impact of protein complexes on diseases, it is to be expected that the composition of individual patients’ complexomes plays a significant role in disease processes. Despite their importance, there has not been a comprehensive investigation of interactions between available drugs and known protein complexes to our knowledge. Numerous individual studies have already described the effects of drugs on protein complexes, motivating us to extend the CORUM database with information on drug effects.

Established databases such as DrugCentral (12), Drug Bank (13), ChEMBL (14) and Guide to Pharmacology (15) provide extensive information on biological activity, drug use, structure and drug targets. Recently, systematic investigations into the druggability of the proteome were initiated. The Illuminating the Druggable Genome (IDG) initiative has established evidence-based criteria for tracking the target development level (TDL) of human proteins (16). In the current CORUM release, we have adopted this classification to create a manageable set of well-characterized protein drug targets.

In the CORUM 5.0 release, we have initiated a systematic and comprehensive investigation of the effects of drugs on protein complexes. The complete dataset has been expanded by 1989 protein complexes, now encompassing 7193 mammalian protein complexes. The newly added protein complexes are biased towards functional complex groups such as G protein-coupled receptors (GPCRs), ion transporters and nuclear hormone receptors with focus on approved drug targets. The CORUM dataset now encompasses 3319 protein complexes containing at least one drug target subunit, potentially influenced by drugs. From 589 annotated protein drug targets, we found at least one interaction between an approved drug and a protein complex, totalling 1975 such interactions. This expanded dataset aims to explain variable drug responses among patients, aid pharmacological studies in early identification of off-target effects and enhance understanding of cellular processes during drug administration.

## Results and discussion

### Extended content of CORUM

Since 2007, CORUM has been a publicly available repository of manually annotated mammalian protein complexes. Compared with the previous CORUM publication (1), the dataset has expanded from 5204 to 7193 protein complexes, representing the most comprehensive database in the field. Other resources covering protein complexes from various organisms are Complex Portal and Reactome (17,18). Regarding the organisms used to characterize these complexes, there are only minor changes compared with the previous publication, with the majority being isolated from human (71%), followed by mouse (15%) and rat (8%). The growth of the CORUM dataset is also reflected in the increased total number of different gene products used as protein complex subunits, rising from 5299 in CORUM 4.0 to 5873. This accounts for 30% of the 19 846 protein-coding genes identified in the human genome assembly GRCh38.p14 (19). A significant portion of this growth is attributed to protein complexes that include protein drug targets as subunits, particularly within the functional complex groups of GPCRs (now 678 complexes), ion channels (now 170 complexes) and nuclear hormone receptors (now 523 complexes).

### Annotation of drug targets in CORUM

To annotate the influence of drugs on protein complexes, we generated a high-quality dataset containing data on protein drug targets. Additionally, we focused on the most thoroughly investigated drug targets to keep the project within a feasible scope. For this purpose, we utilized the classification scheme developed by the IDG Knowledge Management Center (KMC) to classify the druggability of the proteome (16). This TDL classification scheme categorizes proteins into four groups – Tclin, Tchem, Tbio and Tdark – based on the depth of investigation from clinical, chemical and biological perspectives. Tclin (clinic) proteins are drug targets linked to at least one active pharmaceutical ingredient approved by the FDA or other regulatory agencies by mechanism of action (MoA). MoA target annotations provide a mechanistic understanding of drug action at the molecular level and relate protein targets to human diseases and symptoms.

A resource providing such information is DrugCentral. DrugCentral has been a public digital database aggregating drug information since 2016 (20). MoA annotations in DrugCentral are collected from several expert-curated resources [ChEMBL database (14), Guide to Pharmacology (15) and KEGG Drug (21)] and manual curations extracted from drug labels and literature. DrugCentral has become an essential component of the KMC Datasets and Tools (22) in the NIH Common Fund’s IDG consortium (https://commonfund.nih.gov/idg). Our annotation was based on 725 human genome-derived proteins from the January 2023 release, for which there exists an approved drug for humans and for which the MoA is known. These drug targets are associated with 1548 drugs.

We comprehensively search for protein complexes for individual protein drug targets, focussing on human complexes. This refers to protein complexes annotated in previous CORUM releases but apply specifically to new complexes. According to the guidelines from the IDG KMC, if the specific subunit (or subunits) cannot be identified as drug targets, we assign all of them as targets (23).

During the manual annotation of identified complexes, we check for published experiments demonstrating that their composition or activity is influenced by a drug. Such information is presented in text form as a drug comment. This comment not only describes the immediate effect of the drug on the complex but often includes the cellular system used and the type of experiment conducted to determine the drug’s effect. Prodrugs such as estradiol valerate or testosterone undecanoate which are converted in the body into a pharmacologically active drug are annotated like the active drugs. The drug comment can also describe the effects of drugs that are not part of our studied dataset. For instance, the androgen receptor (AR) agonist metribolone (R1881) has been described in 37 cases as a drug that regulates AR complexes. Additionally, the drug comment also covers special cases where, for example, a drug activates the activity of a protein complex, but an increasing amount of a subunit inhibits the activity (see CORUM-ID 9333).

If the effect of an approved drug on a protein complex has been demonstrated, the drug effect is also provided as a formalized, computer-readable comment. The formal comment indicated individually for each drug affecting a complex, is enclosed in square brackets and includes the following elements in the specified order: drug target; complex name; CORUM-ID; drug name; drug activity 1; drug activity 2; PMID [e.g. (NOD2; NLRP1-NOD2 inflammasome; 7885; mifamurtide; activates; complex formation; 18511561)]. ‘Drug activity 1’ describes the mode of action, which can be ‘activates’, ‘inhibits’ or ‘modifies’. ‘Modifies’ is used when the drug can have either an inhibitory or activating effect on the protein complex under different conditions.

As of note, most effects of drugs on protein complexes have been characterized in cell lines or *in vitro*. This does not necessarily imply that such interactions take place *in vivo* in all tissues of the human body. For example, dopamine activates the dopamine D2 receptor/neurotensin type 1 receptor heteromer in HEK293 cells (24). Dopamine as neurotransmitter, acts via five dopamine receptors within the central nervous system (CNS) to regulate movement, reward and other functions through its interaction with the five dopamine receptors and monoamine uptake transporters. However, dopamine infused as an intravenous drug does not penetrate the blood–brain–barrier but exerts significant cardiac and vascular effects by acting preferentially, but not exclusively, on the adrenergic receptors. At higher concentrations, dopamine also acts as an indirect sympathomimetic drug by facilitating norepinephrine release (25).

### Drug target complexes in CORUM

Overall, the CORUM dataset contains 3319 protein complexes, each containing at least one subunit that is part of our dataset of 725 drug targets. Of these drug targets, 405 have been fully annotated in the current CORUM release, and 184 are currently in progress. If a drug target is known to function as a monomer, or if it is unknown whether the target protein is a subunit in a complex, this drug target will not appear in CORUM. For the up to now 558 annotated targets, we found regulatory drug functions for 788 complexes and were able to identify 1975 experimentally validated drug–complex interactions which are described in the formal comment.

In simple cases such as the TNNC1-TNNI3-TNNT2 complex (CORUM-ID: 8768), exactly one complex regulated by one approved drug was identified for one drug target. On the other hand, for the COX2 complex (CORUM-ID 8862), we found 42 experimental evidence for the effects of various drugs. In some instances, a large number of complexes was found for individual drug targets. For example, the AR is a subunit in 97 distinct complexes.

Table 1 shows the annotated complexes and known drug effects for five major target classes (GPCRs; ion channels; enzymes; kinases; and nuclear hormone receptors). It is notable that nuclear hormone receptors, despite having relatively few drug targets, form a large group with 523 complexes, whereas there are comparably few complexes in the enzyme group. This is partly because enzymes such as carbonic anhydrases or cytochromes in some cases are only found as monomeric proteins or homomeric complexes, respectively.

**Table 1.** Overview of important target classes

Target class	GPCRs	Ion channels	Enzymes	Kinases	Nuclear hormone receptors
Protein drug targets	111	129	226	70	18
Annotated as complex with drug target	607	403	702	589	550
Complexes with drug text comments	162	116	308	159	487
Complexes with formal drug comments	108	72	132	63	351

‘Protein drug targets’ are all proteins that have been included in our dataset according to the description in the text. ‘Annotated as complex with drug target’ defines all protein complexes that include at least one of the drug targets as a subunit.

A particularly noteworthy group comprises complexes that incorporate multiple drug targets as subunits. Analysis of the CORUM 5.0 dataset reveals that 962 protein complexes contain at least two drug targets. Of these, 374 complexes are supported by experimental evidence of drug action, as documented by formal drug comments. Studies have shown that the effect of two drugs combined does not necessarily correspond to the sum of their individual effects. Examples have been found where drugs can synergistically affect protein complexes. The RXR agonist LGD1069 (bexarotene) and the peroxisome proliferator-activated receptor-gamma (PPARG) agonist rosiglitazone up-regulated the expression of the S100A2 gene (69x) in A375(DRO) melanoma cells. LGD1069 and the PPARG agonist pioglitazone individually up-regulated S100A2 3.4× and 4.9×, respectively, indicating that the combination was synergistic (26). Other examples show that the addition of a second drug can diminish the effect of the first drug. The AR and estrogen receptor alpha (ESR1) form a protein complex (CORUM-ID: 9246) in CV1 cells. When ESR1 is co-transfected with AR, a 35% decrease in glucocorticoid hormone activity was observed in the presence of both estradiol and metribolone compared with cells treated with metribolone alone (27).

### Protein complexes from important drug target classes

*GPCRs*. GPCRs represent the largest superfamily of cell surface transmembrane receptors encoded by about 800 human genes. It is estimated that ∼34% of marketed drugs are modulators of GPCR function (28,29). Although the GPCRs are functional in their own right as monomers/homomers, the formation of GPCR heteromers results in complexes that may have distinct biochemical properties. This expands the complexity of receptor signalling networks, adds selectivity and specificity to receptor signalling and makes these heteromers promising drug targets (30).

GPCRs can form homomers and heteromers not only with other GPCRs, GPCR heterocomplexes can also involve ion channel receptors, receptor tyrosine kinases, sets of G protein-interacting proteins, ion channels and/or transmitter transporters. CORUM 5.0 includes 241 GPCR-GPCR heterocomplexes. DRD1-DRD2 heteromer (CORUM-ID: 10 657) formation, for example, was found to be increased in human post-mortem striatum of subjects with major depression. Depression and anxiety are more common among females than males, and the DRD1-DRD2 heteromers (CORUM-ID: 7756) were overexpressed in female non-human primate and rat brain, along with higher depressive-like and anxiety-like behaviours (31).

GPCR heterocomplexes have also been shown to participate in learning and short and long-term memory and GPCR heterocomplexes in the CNS have become targets for neurotherapeutics in Parkinson’s disease, schizophrenia, substance use disorder, anxiety and depression (32). Heterocomplex formation takes place in an orchestrated spatio-temporal fashion, yet most studies are limited to experimental or pathological settings so the physiological relevance of many GPCR heteromers remains to be established (28).

*Nuclear hormone receptors*. Nuclear receptors are ligand-activated transcription factors that regulate key functions in reproduction, metabolism, development and physiology. Humans have 48 nuclear receptors, which, when dysregulated, are often linked to diseases. Our dataset includes 18 nuclear hormone receptors from the category Tclin. The therapeutic potential of this field was realized with the demonstration that the anti-estrogen tamoxifen could be used to treat women with breast cancer in an estrogen receptor-dependent manner (33). Nuclear receptors exhibiting classic genomic effects can directly bind DNA as homodimers (e.g. glucocorticoid receptor), heterodimers (e.g. RARA-RXRA complex; CORUM-ID: 8874) or monomers (33). The formation of nuclear hormone receptor complexes is characterized by high modularity, affecting protein interactions within this group. Retinoic acid receptor RXR-alpha (RXRA) is found in complexes with at least 11 other nuclear hormone receptors (AR, NR1H4, PPARA, PPARD, PPARG, RARA, RARB, RARG, THRA, THRB, VDR; CORUM-IDs: 9263, 8924, 8922, 9638, 8923, 8874, 8875, 8876, 8872, 8873, 8932).

**Figure 1.**
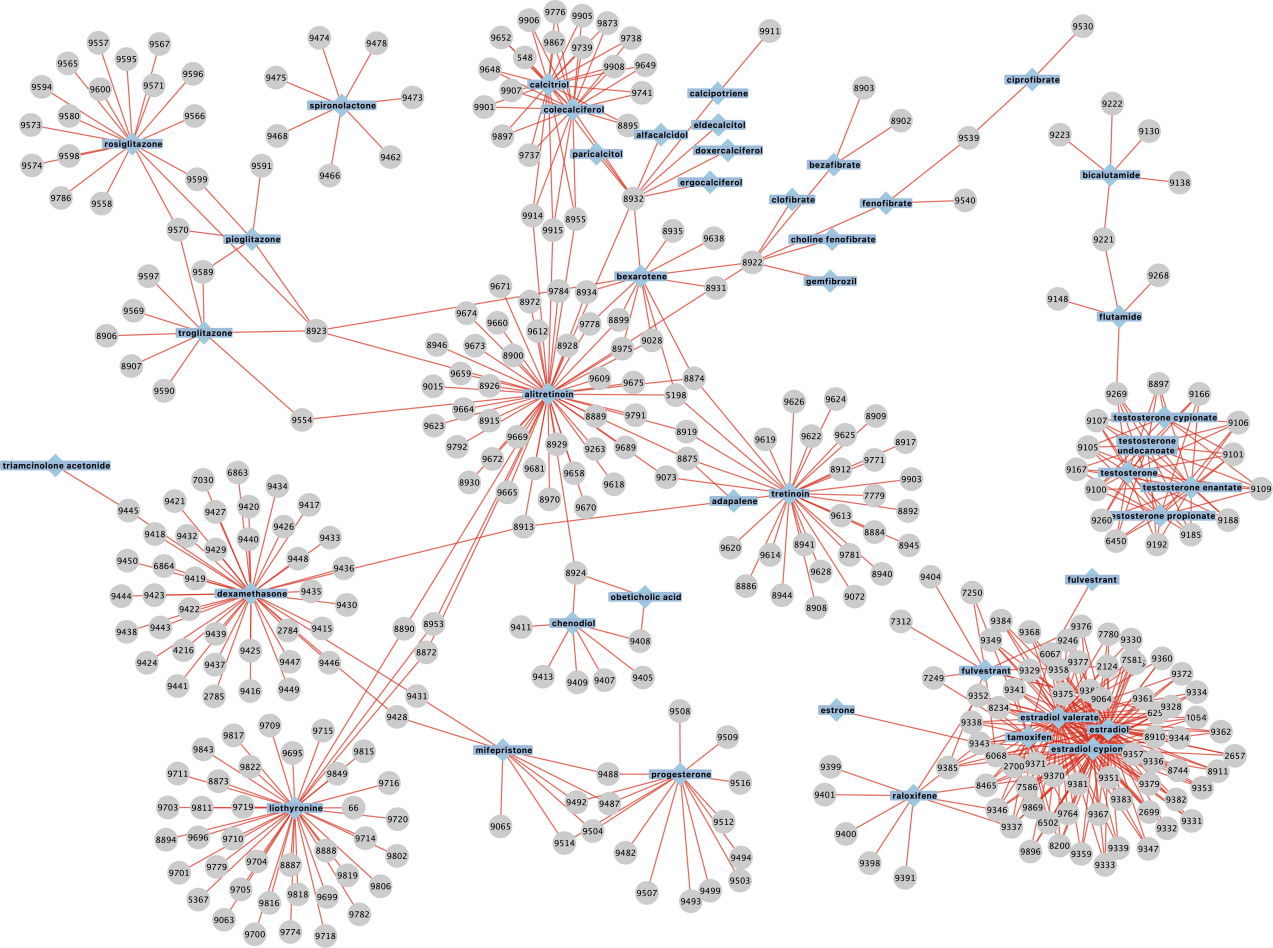
Interaction network of nuclear hormone receptors and their drugs. The graph shows all regulatory interactions between the nuclear hormone receptor protein complexes (grey) in our dataset and the corresponding drugs (blue) based on the formal drug comments.

Another important aspect of understanding nuclear receptor function is the impact of coregulatory proteins that interact with and modulate the transcriptional activity of nuclear receptors (33). Similarly, coregulators tend to regulate more than one nuclear hormone receptor. For instance, the nuclear receptor coactivator 2 interacts with at least 12 nuclear hormone receptors (AR, ESR1, ESR2, NR3C1, PGR, PPARA, PPARG, RARA, RXRA, THRA, THRB, VDR; CORUM-IDs: 8897, 9064, 9400; 9441, 9065, 9534, 9580, 8892, 8893, 8894, 9819, 8895). To date, over 300 AR-interacting proteins have been reported to modulate AR transcriptional activity through several diverse mechanisms (34). The modularity of coregulators explains how the same ligand can regulate different genes in different tissues or developmental stages based on the tissue-specific availability of coregulators (35). The modularity within nuclear receptors and their multiple interactions with various coregulators result in a large group with 523 members interconnected by drugs. Figure 1 shows a graph depicting all 626 experimentally confirmed drug–protein complex interactions of the new CORUM release within the nuclear hormone receptor category. The complexity of the graph points to a key feature of nuclear receptors: their ability to regulate different sets of genes within different cell types based on the tissue-specific transcriptional complexes they form and their ability to select cell-specific enhancers (33).

*Ion channels*. Ion channels mediate communication between cells and their environment. Dysfunctional ion channels are associated with human disorders such as neuronal disorders, diabetes and heart failure (16). Examples show that the function of ion channel complexes is critical for understanding drug action. For instance, the alpha2delta-1 subunit (CACNA2D1), part of voltage-activated Ca2+ channels, binds to gabapentinoids, which are used to treat neuropathic pain and epilepsy. Since CACNA2D1 itself does not affect neuronal function, it was unclear how gabapentinoids work. Recent findings revealed that CACNA2D1 forms a heterodimeric complex with GRIN1 (CORUM-ID: 10 625) and that gabapentin inhibits the formation of the CACNA2D1-GRIN1 complex (36). Neuropathic pain is reduced by inhibiting the forward trafficking of the CACNA2D1-GRIN1 complex. Novel studies have indicated that CACNA2D1 may be a promising drug target not only for neuralgia but also for tumour-initiating cells in gastric cancer, hepatocellular carcinoma and non-small cell lung cancer (37).

Ion channels have also been described where not just one but two subunits are drug targets. An example is the potassium voltage-gated channel KCNQ2-KCNQ3 complex (CORUM-ID: 6712). Mutations in this channel are associated with a rare inherited form of epilepsy known as benign familial neonatal convulsions (38). KCNQ2 and KCNQ3 form a heteromeric potassium channel complex, and the epilepsy drug retigabine can bind both subunits (39). Retigabine functions as an opener of KCNQ2-KCNQ3 K+ channels by markedly shifting the activation voltage to more negative potentials, as well as accelerating the rate of activation and decelerating deactivation (38).

### Understanding drug action in the context of protein complexes

There are multiple examples demonstrating the importance of protein complex information for understanding the effects of drugs. For instance, it has been shown that drugs can have separate responses to the formation of similar complexes. Liver X receptor and PPAR are two members of nuclear receptors involved in the nutrient metabolisms of dietary fatty acids and cholesterol. Using surface plasmon resonance technology, it was shown that the PPARG agonist troglitazone enhances the NR1H3-PPARG heterodimerization (CORUM-ID: 8906) but slightly inhibits the formation of the NR1H2-PPARG complex (CORUM-ID: 8907) (40). Similarly, the non-FDA approved compound LG101506 was identified as a heterodimer-selective RXR modulator that binds specifically to RXR with high affinity and is an RXR homodimer partial agonist. LG101506 was found to selectively activate RXR–PPARA and RXR–PPARG but not RXR–RARA, RXR–NR1H3, RXR–NR1H2 or RXR–FXRA (41).

The knowledge of drug targets as part of protein complexes can also help to understand unexpected effects in the action of drugs. Tumour angiogenesis is a highly complex process mainly regulated by the vascular endothelial growth factor (VEGF) pathway, and precise knowledge of the function of each VEGF factor is crucial for drug development (42). For a long time, the angiogenic effect of the ligand VEGF-B remained unclear, even though drugs that inhibit VEGF-B, along with other members of the VEGF family, are commonly used to treat patients with various neovascular diseases. However, such treatments have shown no benefit in many types of cancer (43). Recently, Lee *et al.* (44) discovered an unexpected function of VEGF-B as an anti-angiogenic factor that prevents fibroblast growth factor 2 (FGF2)-driven angiogenesis and tumour growth when FGF2 and the receptor FGFR1 are abundantly expressed. They showed that VEGF-B promotes the formation of the FGFR1-FLT1-VEGFB complex (CORUM-ID: 10 602), which inhibits excessive angiogenesis by competing with FGF2 for binding to FGFR1. This finding provides one possible explanation for the lack of desired anti-angiogenic effects of VEGF-B inhibitors in some cases.

## Conclusion

With 7193 protein complexes, CORUM provides a broad representation of the protein complex universe in mammals. CORUM 5.0 is the largest publicly available collection of manually annotated mammalian protein complexes, allowing extensive studies of the cellular machines indispensable for most cellular processes. The CORUM dataset is used by a number of external bioinformatics tools and databases and is employed in numerous studies for analysing experimental data (1).

With the current release, CORUM is the only database that systematically annotates the impact of drugs on protein complexes. Numerous examples have demonstrated that the effect of drugs in cells cannot be attributed to their effects on specific drug target proteins. Understanding the diverse interactions of drugs in cells is fundamental to understanding how drugs act in individual patients and why patients may respond differently to the same drugs. Furthermore, this dataset adds another layer of information to network medicine and enables rational drug design in pharmaceutical research. The next goals will be to complete the core dataset of the best-studied drug targets and then expand the dataset to include additional drug targets.

## Data Availability

The provided data can be freely downloaded in various formats from our downloads page (https://mips.helmholtz-muenchen.de/corum/download) under the Creative Commons Attribution License (CC BY 4.0).
